# Nrf2 and autonomic dysregulation in chronic heart failure and hypertension

**DOI:** 10.3389/fphys.2023.1206527

**Published:** 2023-08-31

**Authors:** Ahmed M. Wafi

**Affiliations:** Physiology Department, Faculty of Medicine, Jazan University, Jizan, Saudi Arabia

**Keywords:** sympathoexcitation, oxidative stress, redox homeostasis, heart failure, hypertension

## Abstract

Redox imbalance plays essential role in the pathogenesis of cardiovascular diseases. Chronic heart failure (CHF) and hypertension are associated with central oxidative stress, which is partly mediated by the downregulation of antioxidant enzymes in the central autonomic neurons that regulate sympathetic outflow, resulting in sympathoexcitation. Antioxidant proteins are partially regulated by the transcriptional factor nuclear factor erythroid 2-related factor 2 (Nrf2). Downregulation of Nrf2 is key to disrupting central redox homeostasis and mediating sympathetic nerve activity in the setting of Chronic heart failure and hypertension. Nrf2, in turn, is regulated by various mechanisms, such as extracellular vesicle-enriched microRNAs derived from several cell types, including heart and skeletal muscle. In this review, we discuss the role of Nrf2 in regulating oxidative stress in the brain and its impact on sympathoexcitation in Chronic heart failure and hypertension. Importantly, we also discuss interorgan communication via extracellular vesicle pathways that mediate central redox imbalance through Nrf2 signaling.

## 1 Introduction

Cardiovascular diseases are the leading cause of morbidity and mortality worldwide, posing a huge health and economic burden (Kearney et al., 2005). While death rates from cardiovascular diseases reduced from 2000 to 2010, cardiovascular diseases remain responsible for approximately one in every three deaths in the United States, accounting for more than 17.3 million deaths (approximately 31% of all global deaths) in 2013 and $863 billion in healthcare costs (Tsao et al., 2022).

Although the entire class of cardiovascular diseases comprises different pathologies, including cardiomyopathies, vascular dysfunction, stroke, and arrhythmias, to name only a few, heart failure and hypertension are the two most common forms of cardiovascular diseases (NCD Risk Factor Collaboration NCD-RisC, 2021; Díez and Butler, 2023). Heart failure is a pathophysiological condition in which an abnormality in cardiac function results in failure of the heart to pump blood at a rate that meets metabolic demands of body tissues (Bloom et al., 2017). Symptoms of heart failure include shortness of breath, fatigue, and exercise intolerance, and often also include fluid accumulation in lung, abdomen, and lower extremities. Consequently, heart failure is accompanied by a substantial morbidity and mortality (Yancy et al., 2013).

Heart failure can be divided into heart failure with reduced ejection fraction (HFrEF) and heart failure with preserved ejection fraction (HFpEF). In HFrEF, the ejection fraction (the proportion of blood pumped from the vernicles) is usually ≤40% due to weakened or impaired contractility of the myocardium (Bloom et al., 2017). Conversely, in HFpEF, the left ventricular ejection fraction is preserved, but the chamber cannot adequately fill with blood because the myocardium becomes thickened or stiffed, resulting in diastolic dysfunction. The current review focuses on HFrEF, which will be referred to as CHF (Borlaug, 2014).

The pathophysiology of CHF is complex and usually begins with a precipitating event such as coronary artery disease, myocardial infarction, and dilated cardiomyopathy, resulting in reduced contraction of the ventricles (Bloom et al., 2017). Consequently, a series of compensatory mechanisms are activated to promote an increase in cardiac output and to maintain end-organ perfusion. These compensatory mechanisms include neurohumoral activation of the sympathetic nervous system and the renin angiotensin system (Zucker et al., 2012). Initially, neurohumoral activation is beneficial in maintaining cardiac output; however, chronic activation of these compensatory mechanisms can be maladaptive and may result in pathological ventricular remodeling and a worsening of CHF (Zucker et al., 2012; Bloom et al., 2017).

Hypertension is one of the most significant risk factors for CHF and other causes of death (World Health, 2009). One in every three adults in the United States is estimated to have hypertension (Tsao et al., 2022). Hypertension is the most common comorbidity associated with CHF. Approximately 73% of patients with acute decompensated CHF exhibited hypertension (Adams et al., 2005). Although pathological conditions such as renal artery stenosis, diabetes, and obesity are well-known causes of secondary hypertension, the essential or primary hypertension etiology remains elusive. However, experimental studies have demonstrated neurological components to the pathogenesis of primary hypertension via increased activity of the sympathetic nervous system (Zimmerman and Davisson, 2004; Young and Davisson, 2015).

Before we discuss the role of the Nrf2 in the modulation of central sympathetic nerve activity, it is also necessary to discuss the role of the renin-angiotensin system and reactive oxygen species in central sympathoexcitation in CHF and hypertension. We will therefore provide an overview of these mechanisms before discussing the role of Nrf2 in regulating central sympathoexcitation, including inter-organs communication pathway between the heart and sympathoregulatory areas in the central nervous system via cargos enriched extracellular vesicles (EVs) that modulate Nrf2 activity in the brain.

### 1.1 Renin angiotensin system

The renin-angiotensin system (RAS) plays a critical role in cardiovascular homeostasis, body fluid regulation, and electrolyte balance (Guyton et al., 1972). In CHF and many forms of hypertension, the renin-angiotensin system is hyperactivated. (Zucker and Zimmerman, 2011; Zucker et al., 2012; Biancardi et al., 2014). In brief, the classical RAS is composed of renin, a protease released by juxtaglomerular cells of the kidneys, and angiotensinogen, a precursor synthesized in the liver. Renin cleaves angiotensinogen, generating inactive angiotensin I. Angiotensin converting enzyme (ACE) located on endothelial cells of the lung and kidneys cleaves angiotensin I to generate Angiotensin II (Ang II), the predominant effector protein of the RAS pathway. Binding of Ang II to angiotensin II type 1 receptor (AT1R) results in an increase in blood pressure through several mechanisms including vasoconstriction, increased renal sodium reabsorption, and the release of aldosterone and arginine vasopressin from renal and pituitary glands, respectively, in addition to increasing central sympathetic outflow (Yang et al., 2011; Gao et al., 2014; Sparks et al., 2014).

Angiotensin-converting enzyme 2 (ACE2) is a homolog of ACE that converts Ang II to Ang one to seven, which subsequently acts upon Mas receptors whose actions counterbalance several deleterious effects of Ang II. As such, ACE2 and Ang one to seven have been thought to be protective to the cardiovascular system (Santos et al., 2013).

In addition to circulating Ang II, all RAS components have now been identified in the brain (Nakagawa and Sigmund, 2017). The RAS control of central blood pressure regulation has been extensively reviewed in the current literature, revealing strong evidence for its importance (Coble et al., 2015; de Kloet et al., 2016; Nakagawa and Sigmund, 2017). A number of animal studies have demonstrated the contribution of brain RAS to the development of hypertension (Sinnayah et al., 2006; Li et al., 2012) and heart failure (Nishimura et al., 2007; Gao et al., 2008). In addition, circulating Ang II may also gain access to cardiovascular centers within the central nervous system via disruption of blood-brain barrier, resulting in exacerbated sympathoexcitatory activity; this, in turn, contribute to the progression of heart failure and neurogenic hypertension (Biancardi and Stern, 2016).

### 1.2 Reactive oxygen species and sympathetic outflow in the central nervous system

Reactive oxygen species (ROS) are highly reactive oxygen-containing compounds produced by all aerobic cells (Zimmerman and Davisson, 2004). Normally, ROS generation is under tight homeostatic regulation, as excessive ROS production can lead to DNA methylation, lipid peroxidation, protein damage, and ultimately cell death via necrosis or apoptosis (Carmody and Cotter, 2001; Ueda et al., 2002). As such, several antioxidant enzymes, such as superoxide dismutase, catalase, and glutathione peroxidase, have evolved to defend against changes in the cellular redox environment. When ROS levels exceed the antioxidant defense capacity due to ROS overactivation, dysregulation of ROS scavenging system, or mitochondrial production, a harmful effect known as oxidative stress occurs. It is now well-established that several pathological conditions, including CHF and hypertension are associated with oxidative stress in the cardiovascular system (Landmesser and Harrison, 2001; Sorescu and Griendling, 2002).

In mammalian cells, ROS are mainly generated from the incomplete reduction of molecular oxygen via enzymatic reactions that involve nicotine adenine dinucleotide phosphate NAD(P)H, xanthine oxidase, mitochondrial electron transport chain, uncoupling of nitric oxide synthase, and through the action of cyclooxygenases, and cytochrome P450 reductases (Cohen, 1994; Griendling et al., 2000; Zorov et al., 2014).

Reactive oxygen species in the central nervous system are best known for their association with neurodegenerative diseases and ischemic conditions, such as stroke (Klein and Ackerman, 2003). However, it is now well-accepted that ROS mediates various biological effects on cardiovascular functions (Touyz, 2004), and their role in mediating alterations in the autonomic outflow has become prominent. The increase in central oxidative stress plays a major role in the activation of sympathetic outflow in CHF and hypertension (Zimmerman et al., 2004; Zucker et al., 2009). A bulk of existing data strongly indicates that the Ang II-mediated increase in AT1R expression and, subsequently, the sympathetic outflow is further increased by the production of ROS such as superoxide anion through the action of NAD(P)H oxidase (Gao et al., 2004; Zimmerman et al., 2004).

The elevation of central oxidative stress along with a reduction in antioxidant proteins contribute to either sensitization or activation of certain populations of neurons via alterations in potassium and calcium channels activity (Sun et al., 2005; Zimmerman et al., 2005). In addition, the decrease in the sympathoinhibitory effect of nitric oxide, resulting from both enhanced nitric oxide scavenging and a reduced activity of neuronal nitric oxide synthase in CHF and hypertension, contributes to sympathoexcitation (Feng et al., 2010; Zheng et al., 2011).

### 1.3 Sympathetic nervous system activation

Under normal conditions, the sympathetic tone is necessary to maintain peripheral vascular resistance and arterial pressure for adequate tissue perfusion. The receptors that adjust this sympathetic tone are primarily located in the great vessels (baroreceptors), the heart, and the aortic and carotid bodies (chemoreceptors). Basic and clinical studies have demonstrated that the ability of these receptors to transmit arterial pressure, blood volume, and oxygen tension information is markedly impaired in the setting of CHF and many forms of hypertension (Ellenbogen et al., 1989; Guyenet, 2006; Zucker et al., 2012). Early work revealed that in patients and animals with CHF, the depression of baroreflex gain resulted in sympathoexcitation (Ferguson et al., 1984). The gain of sensory ending receptors in the low-pressure side is also reduced and contributes to sympathoexcitation by removing the inhibitory restraints (Zheng et al., 2006). The input from cardiac sympathetic afferents has also been shown to be augmented in the setting of the CHF (Schultz and Li, 2007).

In addition to cardiovascular sensory dysfunction, various abnormalities in the reflex arc have been shown to contribute to sympathoexcitation. Central changes in the membrane sensitivity of pre-sympathetic neurons at several medullary and hypothalamic sites also contribute to enhanced sympathetic nerve activity (Triposkiadis et al., 2009). The enhanced sympathetic outflow has several effects on innervated visceral organs. Cardiac sympathetic nerves increase myocardial oxygen demands by releasing norepinephrine, which acts on myocardial adrenergic receptors. This results in increased Ca^+2^ signaling, thus increasing contractility and myocardial oxygen demand. β1 adrenergic receptor inhibitors, such as propranolol, are often used clinically to reduce sympathetic heart tone in order to decrease cardiac metabolic demand. Renal sympathetic nerve activity causes afferent arteriolar constriction, resulting in decreased glomerular filtration rate, high sodium and water retention, and renin release, thus stimulating the RAS activity (Hall, 2016). Increased vascular sympathetic tone results in a robust increase in vascular resistance and blood pressure (Zucker et al., 2012). The central mechanisms underlying this increase in sympathetic nerve activity are important to understand for the future management of cardiovascular diseases.

### 1.4 Central autonomic control areas

The central neuronal networks that contribute to cardiovascular and sympathetic regulation are distributed throughout the brain in the brain stem, hypothalamus, and forebrain (Guyenet, 2006; Guyenet et al., 2020). These networks are comprised of circuits through which peripheral afferent neurons terminate in the nucleus tractus solitarius (NTS). Excitatory neurons, mostly glutamatergic, project to the caudal ventrolateral medulla (CVLM) and evoke inhibitory neurons (GABAergic) to the rostral ventrolateral medulla (RVLM). The RVLM neurons have direct projections to the intermediolateral column of the spinal cord, where they excite the sympathetic preganglionic neurons and, ultimately, end organs sympathetic nerves (Llewellyn-Smith and Verberne, 2011). Thus, the RVLM is a key mediator of vasomotor tone. Nearly all vasomotor tone is removed after pharmacological inhibition or ablation of RVLM (Schreihofer et al., 2000). Paraventricular nucleus (PVN) neurons also have direct projections to sympathetic preganglionic neurons and the RVLM (Zheng and Patel, 2017). Therefore, the PVN can directly influence vasomotor tone via direct projections to sympathetic preganglionic neurons or the RVLM.

Neurons in the RVLM are susceptible to increased ROS levels. Increased sympathetic outflow in CHF and several hypertension animal models has been attributed to oxidative stress in the RVLM (Zimmerman and Davisson, 2004; Sun et al., 2009).

## 2 Keap1-Nrf2 signaling pathway

**FIGURE 1:**
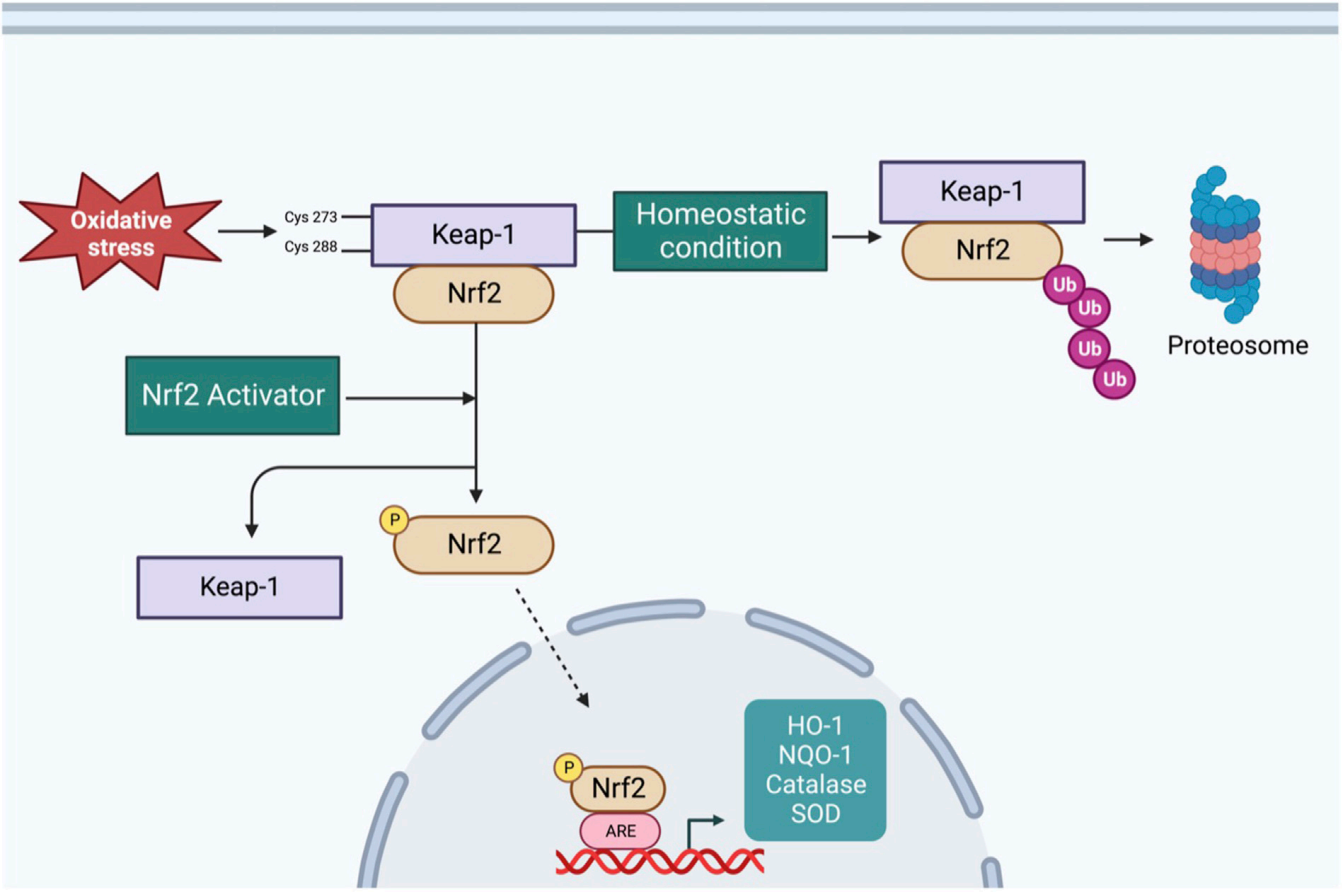
Schematic overview of Nrf2 signaling pathway–Under basal condition, Nrf2 is tethered to Keap-1, resulting in polyubiquitination and proteasomal degradation of Nrf2. Under oxidative stress condition, cysteine residues of Keap-1 are oxidized, resulting in a conformational change and translocation of Nrf2 into nucleus, where it binds to ARE to increase the transcription of antioxidant enzymes. Additionally, varieties of phytochemicals can activate Nrf2 through Keap-1 independent mechanism via phosphorylation by different kinases.

The redox-sensitive transcription factor Nrf2 and its tether Kelch-like ECH-associated protein 1 (Keap1) play important roles in maintaining redox homeostasis in various types of cells, including neurons (Cuadrado et al., 2019). Figure 1 shows a schematic of the Nrf2 signaling pathway. Under normal conditions, Nrf2 is primarily found in the cytoplasm in conjunction with Keap-1, which forms a ubiquitin E3 ligase with Cullin3 and polyubiquitinates Nrf2 for degradation via the proteasome system (Yamamoto et al., 2018). Therefore, under unstressed conditions, Nrf2 is synthesized but constantly degraded. However, under oxidative stress, the sulfhydryl group of cysteine residues in Keap-1 is oxidized, resulting in the dissociation of Nrf2 from the Keap-1 cullin3 complex. Once Nrf2 is released from Keap-1, it translocates into the nucleus, where it binds to antioxidant response elements (ARE) to upregulate the expression of several antioxidant enzymes (Yamamoto et al., 2018). Since Nrf2 is a master regulator of antioxidant enzymes expression, it is logical to assume it would protect against ROS-related cardiovascular diseases such as CHF and hypertension. In the following sections, we summarize the role of RVLM Nrf2 in the modulation of sympathetic outflow in CHF and hypertension.

### 2.1 Is Nrf2 in the brain associated with sympathetic nerve activity?

The transcription factor Nrf2 is a master regulator of several antioxidant enzymes in many cell types, including neurons. Current evidence suggests that oxidative stress is a potent mediator of enhanced sympathetic outflow in CHF and hypertension settings (Zimmerman and Davisson, 2004). While a component of oxidative stress is mediated by pro-oxidant signaling such as Ang II, norepinephrine, and cytokines (Patel and Zheng, 2012), there is a reduction of antioxidant enzymes production that contribute to excessive ROS in the central nervous system of animals with CHF and hypertension (Gao et al., 2007). A reduction in Nrf2 protein levels or its retention in the cytosol will reduce the influence of this transcription factor on the ARE and, subsequently, the expression of the antioxidant enzymes.

Indeed, in the RVLM of animals with CHF, high norepinephrine levels are associated with reduced Nrf2 mRNA and protein expression. Interestingly, normalized Nrf2 expression in whole cell lysates of the RVLM showed a correlation with ejection fraction or cardiac function for both protein and mRNA (Wafi et al., 2019). While this association does not prove a cause-effect relationship, it points to a strong possibility that these two parameters may be somehow related and may also indicate a regulatory mechanism with some sort of feedback from either local oxidative stress in the central neurons mediated by the metabolic activity of these neurons, or communication between the periphery and the central nervous system. In hypertensive rats induced by lipopolysaccharide, reduced Nrf2 protein expression in the RVLM neurons leads to impaired mitochondrial biogenesis and contributes to the development of hypertension (Wu et al., 2016). Furthermore, in the PVN of spontaneous hypertensive rates, both cytosolic and nuclear Nrf2 are reduced (Bai et al., 2017).

A variety of mechanisms contribute to the reduced Nrf2 levels in the RVLM in CHF and hypertension. The studies mentioned above clearly demonstrated a reduction in Nrf2 transcription. In addition, enhanced Nrf2 ubiquitination and nuclear factor kappa-light-chain-enhancer of activated B cells (NFKB) signaling may also contribute to the reduced Nrf2 levels. Nrf2 binds to the nuclear protein CREB Binding Protein (CBP) in a similar fashion to NFKB. Because NFKB and Nrf2 are redox-sensitive transcription factors, the competition for binding to CBP may be a key regulator of oxidative stress in the central neurons involved in the regulation of sympathetic outflow. Liu et al. have shown that Nrf2 displaces NFKB from CBP through competitive interaction with a specific Creb Binding Protein (CBP) domain (Liu et al., 2008). Importantly, activation of Nrf2 by bardoxolone methyl reduced NFKB binding and increased Nrf2 binding to the nuclear CBP in the myocardium of CHF rats (Tian et al., 2019). Whether a similar mechanism can be extended to central autonomic neurons has yet to be determined. Another mechanism for which there is a growing body of evidence is the influence of extracellular vesicles and their cargo on the translation of Nrf2 in remote organs, including the brain. This will be discussed further in the subsequent sections.

### 2.2 Does Nrf2 knockdown or overexpression in the RVLM alter oxidative stress, sympathetic nerve activity, baroreflex function, and blood pressure?

**FIGURE 2:**
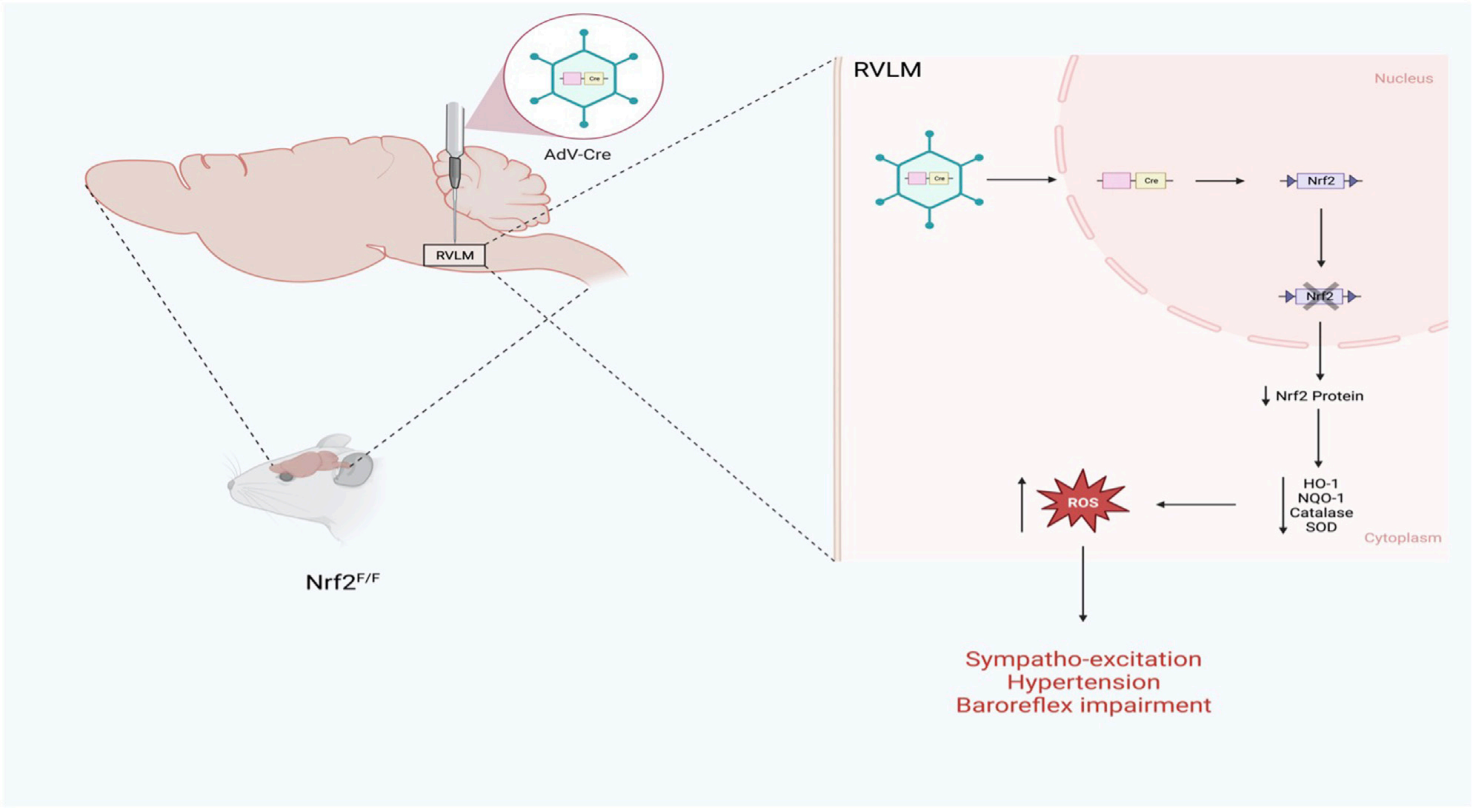
Schematic representation of the impact of Nrf2 gene deletion selectively in the RVLM of normal mice on sympathetic outflow and blood pressure (Based on the findings of Gao et al.) (Gao et al., 2017). Nrf2 gene deletion in the RVLM increases blood pressure, augments sympathetic outflow, and impairs baroreflex function.

The role of Nrf2 as a mediator of sympathoexcitation in CHF and in response to ANG II has recently been demonstrated in animal models (Gao et al., 2017; Ma et al., 2019). Downregulation of antioxidant enzymes in sympatho-regulatory areas contributes, in part, to increased ROS levels. Given the role of oxidative stress modulation of ion channels and enhanced sensitivity of pre-sympathetic neurons (Gao et al., 2007), and because Nrf2 is a master regulator of antioxidant enzymes expression (Cuadrado et al., 2019), the sympaltho-excitation is likely mediated by reduced Nrf2 levels in central autonomic areas. Indeed, in the RVLM of Nrf2 floxed mice, Nrf2 gene deletion using lentiviral Cre RVLM microinjection resulted in a remarkable reduction in Nrf2 mRNA and protein level, leading to a reduction in antioxidant enzymes and elevation of ROS levels as illustrated in Figure 2 (Gao et al., 2017). While one may assume that the level of Nrf2 in normal animals is relatively low if they were not under oxidative stress, and as such, there would not be much changes in sympathetic outflow, the above mouse model displayed sustained hypertension and showed approximately 20%–25% increase in the mean arterial pressure suggesting that the basal level of Nrf2 in the normal state is crucial for restraining sympathetic outflow. Although immunofluorescence data clearly demonstrated that the RVLM neurons were successfully targeted by the lentiviral Cre, neuronal subtypes cannot be discriminated. Furthermore, the potential role of glial cells in the detrimental maladaptive increase in sympathoexcitation observed in these Nrf2-deficient mice cannot be excluded. Although the functional implications of ROS are lacking, the link between altered glial cell activity and the excitability of pre-sympathetic neurons has been recently demonstrated (Marina et al., 2016).

**FIGURE 3:**
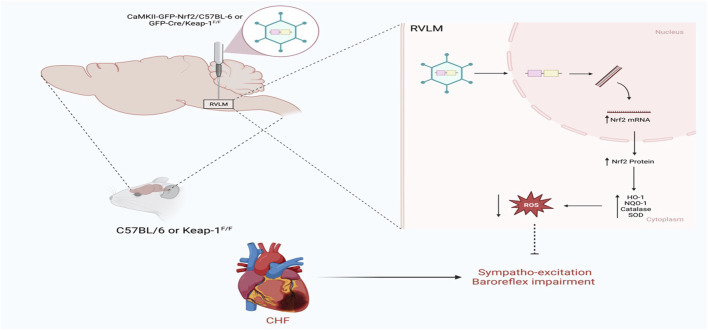
Schematic representation of the impact of Nrf2 upregulation in the RVLM on sympathetic outflow in CHF state using either C57BL/6 or Keap-1^f/f^ mice (Based on the findings of Ma et al.) (Ma et al., 2019). In C57BL/6, Nrf2 was overexpressed in the RVLM using HIV-CamKII-Nrf2 Lenti virus, whereas in Keap-1^f/f^ mice, Nrf2 was overexpressed in the RVLM using Lenti-Cre virus. Nrf2 upregulation selectively in the RVLM ameliorates sympathoexcitation in CHF mice.

The other side of the coin is what happens if Nrf2 is selectively overexpressed in the RVLM in a disease setting. This question has recently been addressed using two mouse models, as shown in Figure 3. In the first model, the RVLM of C57BL/6 CHF mice was transfected with the Nrf2 gene driven by the CaMKII promoter to selectively transfect the glutamatergic neurons; in this way, either tyrosine hydroxylase positive neurons or glutamatergic neurons can be targeted for Nrf2 overexpression (Dittgen et al., 2004). The other model for selective Nrf2 overexpression in the RVLM involved Keap-1 floxed mice, in which the RVLM was microinjected with lentiviral Cre. The data from these two models (Ma et al., 2019) clearly indicates that when Nrf2 was overexpressed in the RVLM, antioxidant enzyme expression increased; was associated with low plasma norepinephrine levels, improved baroreflex function, and reduced sympathetic nerve activity. However, cardiac function did not improve in these animals, probably due to the massive infarct size of the left ventricle in these mice.

### 2.3 Does exercise training reduce sympathetic nerve activity by Nrf2-dependent mechanisms?

Substantial evidence now indicates that ExT can normalize the autonomic imbalance, especially in the setting of CHF. Clinical trials have shown that ExT increases survival and reduces cardiovascular events and hospitalization in patients with CHF (O’Connor et al., 2009; Downing and Balady, 2011). Varieties of central mechanisms are responsible for sympathoinhibition following ExT in CHF. These mechanisms include increased neuronal nitric oxide synthase (nNOS) (Sharma and Patel, 2017), increased GABAergic tone, decreased glutamate and NMDA signaling (Kleiber et al., 2008; Wang et al., 2013), a reduced AT1R-oxidative stress axis, and increased antioxidants enzymes expression (Gao et al., 2007; Kar et al., 2010). The link between ExT and the changes in the central antioxidant enzyme involves the Nrf2-keap-1 signaling pathway. ExT has been shown to upregulate Nrf2 mRNA and protein expression in the RVLM of CHF animals, which was associated with the upregulation of antioxidant enzymes and a reduction in plasma norepinephrine levels (Wafi et al., 2019).

Interestingly, in the RVLM, Nrf2 expression varies as a function of cardiac function, independent of the sedentary and exercise status of animals (Wafi et al., 2019). This may depict a continuum of antioxidant regulation in sympathetic neurons that is driven, in part, by cardiac function, i.e., cardiac output. These data, however, did not reveal whether the reduction in sympathetic nerve activity in response to ExT in the CHF state is Nrf2-dependent. The relationship between central Nrf2 expression and sympathetic nerve activity in response to ExT can only be conclusively tested by selectively knocking down Nrf2 in the RVLM of animals with CHF. This will provide critical evidence of a cause-effect relationship.

Although it is generally accepted that the benefits of ExT in CHF rely, in part, on the upregulation of central antioxidant enzymes, it is not completely clear how the act of ExT is transduced into a change in antioxidant enzymes expression in the brain. In this regard, three possibilities have been proposed. First, the increased metabolic activity of the pre-sympathetic neurons during exercise allows these neurons to cope with oxidative stress. For instance, ROS generated during skeletal muscle contraction provides a circulating stimulus to remote tissues, including the brain stem, triggering an adaptive response to upregulate antioxidant enzymes, potentially via Nrf2. A second intriguing pathway that may be important in the setting of CHF, hypertension, and other sympathoexcitatory disorders is the modulation of the balance between the redox-sensitive transcription factors Nrf2 and NFKB. Although the interaction between these transcription factors in sympathoregulatory areas of the brain has never been explored, we speculate that in CHF and hypertension, Nrf2 and NFKB CBP-binding competition may be an important pathway. A third possibility is the notion that extracellular vesicles (EVs) shed from exercising muscles can influence remote tissues such as the brain stem, where EVs and their enzymatic and non-enzymatic cargo can regulate antioxidant pathways, potentially via the Nrf2 signaling pathway.

### 2.4 Does ACE2 reduce sympathetic nerve activity via Nrf2-dependent mechanisms?

Angiotensin-converting enzyme 2 is a carboxypeptidase expressed in the brain stem and hypothalamic brain areas and contributes to regulating blood pressure and peripheral resistance by modulating the sympathetic outflow (de Morais et al., 2018). Because ACE inhibition is a primary therapy for patients with CHF (Zucker and Zimmerman, 2011), the role of ACE2 and its metabolites, including Ang1-7, becomes more prominent. Several studies have implicated ACE2 and Ang1-7 as important modulators of sympathetic outflow and the neurogenic control of blood pressure. In rabbits with CHF (pacing-induced), the central ACE is increased, and ACE2 is decreased in the RVLM, NTS, and PVN (Kar et al., 2010). Selective overexpression of ACE2 in the brain of CHF mice resulted in the downregulation of AT1R in the RVLM (Xiao et al., 2011). Sriramula et al. clearly showed that selective overexpression of ACE2 in the brain reduces the pressor response to chronic Ang II infusion (Sriramula et al., 2011). The mechanism by which ACE2 ameliorates pressor response and sympathoexcitation in CHF and hypertension is mediated, in part, by the reduction in the central oxidative stress (Xia et al., 2011). Since ACE2 can act as an antioxidant, an antioxidant defense mechanism may play an intermediate role. Thus, Nrf2 may at least partially mediate the sympathoinhibitory response to ANG II-induced hypertension.

The potential interaction between ACE2 and Nrf2 in modulating sympathetic nerve activity was recently demonstrated in a synapsin human angiotensin-converting enzyme 2 (syn-hACE^+/+^) mice model. In this model, the human ACE2 gene (*hACE2*) is controlled by the synapsin promoter to induce human ACE2 expression in the brain (Jia et al., 2020). Intracerebroventricular (ICV) Ang II-induced hypertension was attenuated in syn-hACE^+/+^ mice. Although syn-hACE^+/+^ mice showed an increased RVLM Nrf2 expression, ICV Ang II administration did not further increase Nrf2 expression in these mice (Ma et al., 2020). The failure of ANG II to trigger an increase in Nrf2 expression in the RVLM of synhACE^+/+^ mice is difficult to explain since ACE2 and Ang II are expected to have a synergistic effect on Nrf2 expression. Nrf2 is a redox-sensitive transcription factor that could be activated by Ang II-induced ROS formation. However, ACE2 may consume Ang II rapidly before exerting its Nrf2-inducing effect via increased ROS levels. These data indicated that Ang II and ACE2 separately upregulated Nrf2 in the RVLM, and Ang II and ACE2 have no synergistic effect on Nrf2 expression in the RVLM.

Although these data indicate that Nrf2 expression in the RVLM modulates the pathogenesis of Ang II-induced neurogenic hypertension, it is not yet clear whether Nrf2 conclusively mediates the sympathoinhibitory effect of ACE2 in response to central Ang II infusion. Experiments employing selective knocking down of Nrf2 in the RVLM of animals that overexpress ACE2 are key to determining a cause-effect relationship.

### 2.5 Does the heart cross-talk with the brain to regulate Nrf2 and sympathetic nerve activity?

This section discusses the regulation of Nrf2 translation via the delivery of EVs that contain miRNAs through circulation. In particular, we will discuss the role of cardiac-derived EVs in evoking oxidative stress and sympathoexcitation in CHF by disrupting the Nrf2 signaling pathway in pre-sympathetic neurons of RVLM.

Accumulating evidence has shown that, in addition to neuronal communication, the heart can communicate with the brain via biochemical mechanisms (Hodes and Lichtstein, 2014; Havakuk et al., 2017). In this regard, miRNA-enriched EVs have emerged as regulators of paracrine signaling and cell-cell communication in physiological and pathological states by regulating the transcription of proteins in remote organs (Martins-Marques et al., 2021; Gabisonia et al., 2022). Bioinformatics analyses have revealed that miRNAs, including miRNA-27a, miRNA 28a, and miRNA 34a, can target the 3’ UTR of Nrf2 mRNA and suppress its translation (Li et al., 2011; Wang et al., 2022). In rats with CHF, the Nrf2 protein and its downstream antioxidant proteins are downregulated in the heart. Paradoxically, Nrf2 transcription is upregulated, suggesting a regulatory mechanism that reduces the translation of Nrf2 mRNA level in the myocardium. Indeed, three miRNAs were highly expressed in the non-infarcted area: miRNA 27a, miRNA 28-3p, and miRNA 34a (Tian et al., 2018). Gerald Dornll et al. demonstrated that myocardium specimens of CHF patients with left ventricular-assisted devices showed marked changes in miRNAs expression profile, including miRNA 27a. Furthermore, plasmas and myocardial expression of miRNAs that target Nrf2 mRNA are increased in patients with ischemic heart disease on LVAD (Matkovich et al., 2009). Consistently, in cultured cardiac myocytes, the TNF-induced increase in Nrf2 translation is inhibited by miRNAs 27a, miRNA 28-3p, and miRNA 34a mimics (Tian et al., 2018), suggesting that Nrf2 translation inhibition may be mediated, in part, by the upregulation of these Nrf2-related miRNAs. Importantly, in the RVLM of CHF animals, Nrf2 protein expression is downregulated, while the same Nrf2-related mRNAs are increased (Tian et al., 2022).

A previous study has demonstrated that miRNAs targeting Nrf2 are enriched in extracellular vesicles isolated from cultured cardiac myocytes and fibroblast media under cardiac stress induced by TNF alpha (Tian et al., 2018). Electron microscopy and NanoSight analyses revealed a typical size and distribution profile of the exosomes released from cardiac myocytes and fibroblasts. Furthermore, in cocultured labeled fibroblast and myocytes, TNF alpha stimulation increased the uptake of fibroblast-released exosomes into cardiac myocytes (Tian et al., 2018). This exosomes cross-talk between cardiac fibroblasts and myocytes in the cocultured system reduced Nrf2 translation. In circulation, the miRNAs that target Nrf2 were significantly higher in EVs derived from the plasma of CHF rats than those from sham rats (Tian et al., 2022).

**FIGURE 4:**
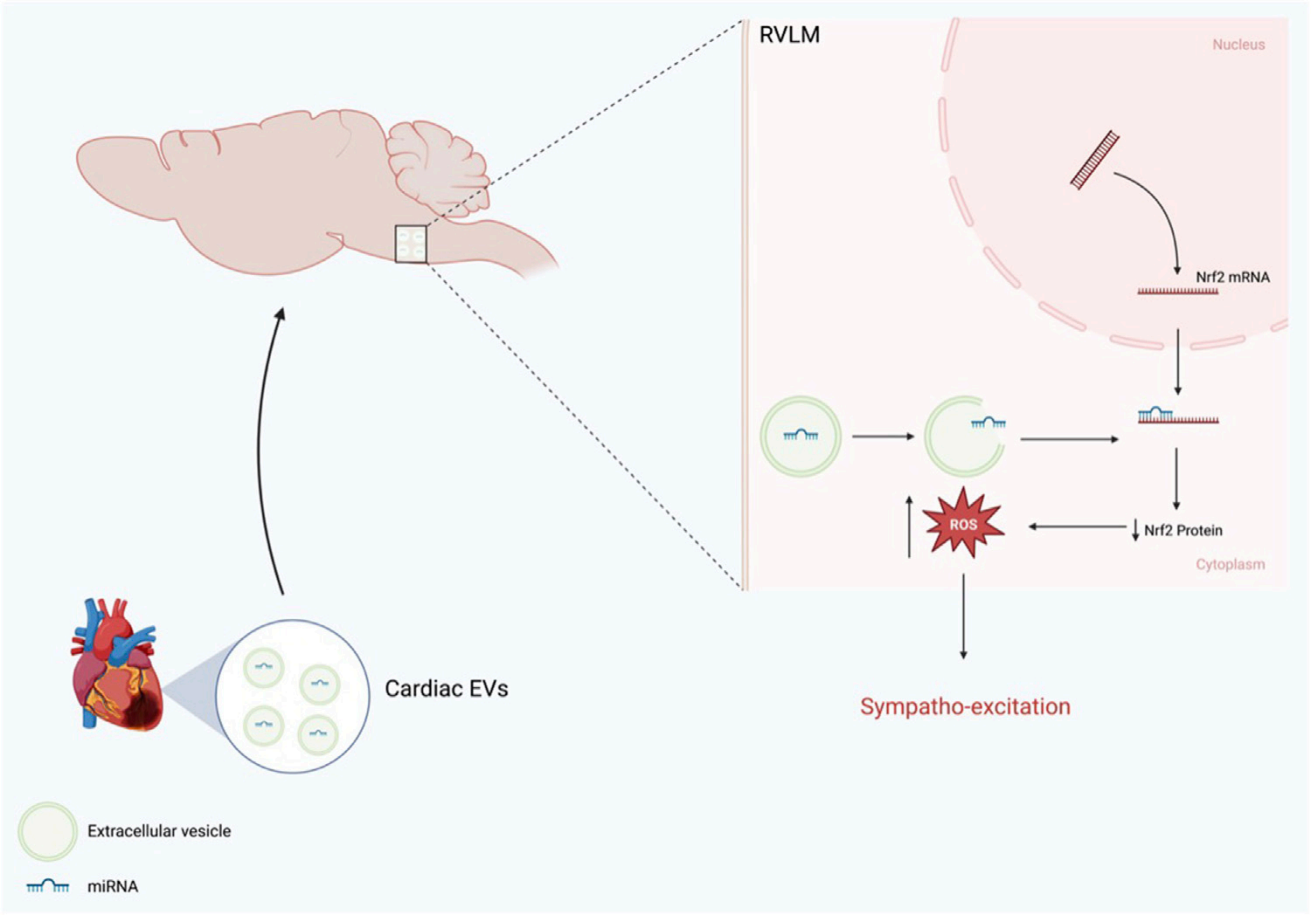
Schematic representation of heart-brain communication via EVs enriched miRNA that modulate Nrf2 expression in the RVLM (Modified from Tian et al.) (Tian et al., 2022).

Circulating EVs seem to pass through the blood-brain barrier and are taken up by RVLM neurons (Tian et al., 2022). *In Vivo* (IVIS) imaging after administration of isolated and labeled EVs into normal mice via intraperitoneal injection revealed their uptake by some organs. However, the brain appeared to concentrate these exosomes after 24 h. Histological examination of RVLM revealed that RVLM neurons take up these EVs, and the distribution of these EVs seemed to be concentrated in the perinuclear region within the neuronal cells. Furthermore, EVs isolated from the brain stem of CHF rats contain not only Nrf2 targeting miRNA but also other miRNAs that are thought to be cardiac specific, including miRNA-1, miRNA 208a, and miRNA-499 (Chistiakov et al., 2016; Huang et al., 2021), suggesting that they are transported from the heart into the brain. To identify the precise origin of the brain EVs, a dual red-green reporter mice model was crossed with myosin heavy-chain tamoxifen-driven transgenic mice to track green particles from the heart to other tissues. In this mouse model, cardiac myocytes exclusively express green fluorescence, whereas all other tissues express a red fluorescent protein. Indeed, histological analysis revealed that green particles were more intense in the brainstem of CHF mice (Tian et al., 2022), suggesting that these particles were derived from the heart. Figure 4 shows a schematic overview of the heart-brain communication pathway that alters Nrf2 signaling pathway in the RVLM.

The role of cardiac-derived EVs in sympathoexcitation has been demonstrated via the transference of pathological phenotypes of CHF rats into normal rats. Microinjections of labeled EVs harvested from CHF rats into the RVLM of normal rats resulted in increased plasma norepinephrine, increased RSNA, and baroreflex impairment (Tian et al., 2022). Interestingly, when these harvested EVs were preloaded with antagomir targeting Nrf2-miRNAs, these effects were attenuated, suggesting that Nrf2-targeting miRNAs may be a potential therapeutic target to ameliorate sympathoexcitation in CHF.

## 3 Clinical perspectives and future considerations

Several animal studies have demonstrated that Nrf2 activation is beneficial in the setting of CHF and Hypertension. Nrf2 activation in the skeletal muscle of coronary artery ligation-induced CHF mice improves exercise capacity (Downing and Balady, 2011). In addition, the activation of Nrf2 by bardoxolone methyl improved cardiac function in a rodent heart failure model (Tian et al., 2019). Furthermore, selective activation of Nrf2 in the RVLM by sulforaphane attenuated the pressor response induced by the central Ang II administration (Ma et al., 2020). These studies suggest that Nrf2 activation may be useful for alleviating sympathoexcitation in the state of CHF and hypertension.

Although antioxidant-based therapies seem to mitigate sympathetic overdrive in animal models of cardiovascular disease, most clinical trials that have attempted to examine the therapeutic effect of antioxidant vitamin therapy in cardiovascular disease have failed. The reason for these failed trials is not completely clear. However, it may relate to facts that these trials targeted only one oxidant species using one antioxidant, or the antioxidant vitamins may not have scavenged the specific ROS driving the pathological conditions. Because Nrf2 is an upstream transcription factor for a variety of antioxidant enzymes and given the detoxification and anti-inflammatory effects of proteins encoded by Nr2 target genes, Nrf2 activator trials in cardiovascular disease may provide promising redox-based therapy. For instance, in patients with mitochondrial myopathy, the Nrf2 activator, omaveloxolone, improved mitochondrial function and submaximal exercise tolerance (Madsen et al., 2020), which could be of great value in the daily lives of patients with CHF. Whether this beneficial effect is mediated by antioxidant or anti-inflammatory mechanisms has yet to be determined.

While Nrf2 activation protects against various cardiac insults, growing evidence has shown a detrimental role of Nrf2 in pathological cardiac remodeling and dysfunction. In pressure overload-induced cardiac injury, cardiac autophagy is impaired, which activates Nrf2-driven angiotensinogen expression, contributing to cardiac pathological remodeling and dysfunction (Narasimhan et al., 2020; Zang et al., 2020). Furthermore, evidence has demonstrated that activation of Nf2 signaling evokes reductive stress, leading to pathological cardiac remodeling and diastolic dysfunction. These results may explain the increased rate of heart failure and deaths and, consequently, the early termination of clinical phase three testing of bardoxolone methyl, a potent Nrf2 activator, in chronic renal disease associated with type 2 diabetes (de Zeeuw et al., 2013; Chin et al., 2014).

In summary, there is robust evidence that Nrf2 contributes to the regulation of oxidative stress in the RVLM and impacts sympathoexcitation in CHF and hypertension. Studies using genetic models clearly demonstrated that the RVLM knockdown of Nrf2 exacerbates sympathetic nerve activity and oxidative stress in CHF and in response to central Ang II administration, while the RVLM overexpression of Nrf2 ameliorates sympathetic nerve activity in CHF. In addition, EV-mediated heart-brain communication evokes sympathoexcitation in the RVLM through miRNAs targeting Nrf2 signaling in CHF.

The role of Nrf2 as a mediator of ExT-induced reduction of sympathetic nerve activity in CHF is speculative and necessitates further mechanistic investigations. However, given the high Nrf2 expression in the RVLM of CHF mice following ExT, Nrf2 could potentially mediates the upregulation of antioxidant enzymes, thereby reducing oxidative stress and attenuating sympatho-excitation in the setting of CHF. Similarly, higher Nrf2 expression in the RVLM of syn-hACE^+/+^ supports the notion that Nrf2 may at least partially mediates ACE2-induced reduction of blood pressure in response to central Ang II administration.

A previous study has demonstrated that the cardioprotective effect of ExT following ischemic-reperfusion injury in mice was abrogated when Nrf2 gene was selectively removed from their skeletal muscle (Gao et al., 2021). Given that skeletal muscle-derived EVs can be transferred into the heart, it will be of interest to determine if skeletal muscle knockdown of Nrf2 would impair cardiac function in the setting of CHF. In addition, the interplay between endothelial dysfunction and oxidative stress has significant implication for blood pressure regulation (Taniyama and Griendling, 2003). Therefore, Nrf2 may be a potential therapeutic target to protect against endothelial dysfunction. It will be of interest to determine whether Nrf2 gene deletion selectively in endothelial cells would modulate blood pressure at rest and in response vasoconstriction induced by Ang II.
